# Virtual reality experiences promote autobiographical retrieval mechanisms: Electrophysiological correlates of laboratory and virtual experiences

**DOI:** 10.1007/s00426-020-01417-x

**Published:** 2020-09-15

**Authors:** Joanna Kisker, Thomas Gruber, Benjamin Schöne

**Affiliations:** Experimental Psychology I, Institute of Psychology, Osnabrück University, Seminarstraße 20, 49074 Osnabrück, Germany

## Abstract

Recent advancements in memory research indicate that virtual reality (VR) experiences are more vividly memorized as compared to conventional laboratory events. In contrast to the latter, VR experiences are highly immersive, simulating the multimodality, vividness and inclusiveness of real-life experiences. Therefore, VR might enable researchers to identify memory processes underlying events which participants have actually experienced, in contrast to conventional on-screen experiences. To differentiate the electrophysiological correlates of memory processes underlying VR experiences as compared to conventional laboratory experiences, participants watched videos either in a PC condition or in a VR condition, followed by an unannounced recognition memory test. As hypothesized, we replicated the well-established theta old/new effect for the PC condition, but remarkably, this effect was absent in the VR condition. Additionally, the latter was accompanied by significantly lower alpha activity as compared to the PC condition. As increases in theta-band responses are related to top-down control on, and memory load during retrieval, the observed theta responses might rather relate to retrieval effort than to retrieval success per se. Congruently, higher alpha activity measured over occipital sensor areas in the PC condition reflect visually guided search processes within episodic memory. The VR condition comes in with lower alpha activity, reflecting immediate and effortless memory access. Hence, our findings indicate that the retrieval of VR experiences promotes autobiographical  retrieval mechanisms, whereas recalling conventional laboratory events comes in with higher effort, which might not reflect the mechanisms of everyday memory.

**Electronic supplementary material:**

The online version of this article (10.1007/s00426-020-01417-x) contains supplementary material, which is available to authorized users.

## Introduction

How people behave in everyday life strongly depends on previous experiences either with a particular situation or personal general knowledge, e.g. concerning the realization of own goals, acting effectively and relating to other peoples (see Conway, 2005). This kind of information is predominantly encoded in and retrieved from autobiographical memory (AM). Similar to episodic memory (EM), autobiographical engrams encode personally experienced events in their respective spatial and temporal context (Tulving, 1983). Extending well beyond EM, AM encompasses highly self-relevant information, especially beliefs and knowledge about the self, experienced events and their relevance (see e.g. Conway, 2005; Greenberg & Rubin, 2003). Hence, AM comprises episodic engrams, extending it by self-referential and emotional processes. The retrieval of autobiographical memories is therefore not limited to temporal, spatial or contextual information, but bears great personal significance (Svoboda, McKinnon, & Levine, 2006). The retrieval of such everyday memories promotes the re-experience of the associated emotions (Svoboda et al., 2006), coming in with vivid and conscious reliving, and foremost the belief that they have actually occurred (Rubin, Schrauf & Greenberg 2003; Greenberg & Rubin, 2003).

While it is common practice to investigate everyday memory in the laboratory using paradigms that induce micro-events prior to recognition memory tests (see Cabeza et al., 2004), these settings are often criticized for lacking the complexity and variety of stimuli and response options characteristic to real-life experiences (Pan & Hamilton, 2018; Kvavilashvili & Ellis, 2004). Specifically, self-relevance and self-involvement are rarely realized in laboratory settings (see e.g. McDermott, Szpunar, & Christ, 2009). Obviously, such traditional approaches face a trade-off between high experimental control and ecological validity, i.e. the validity of the results obtained in the laboratory and generalized to everyday life (see Parsons, 2015).

Potentially overcoming this gap between experimental control and ecological validity, virtual reality (VR) has gained interest as a methodical tool in psychological research (see e.g. Parsons, 2015; Pan & Hamilton, 2018; Schöne et al., 2017, Kisker, Gruber, & Schöne 2019a, b). For memory research, VR experiences might provide a closer approximation to real-life experiences as compared to conventional laboratory settings. The former is characterized by a high level of sensory cues and thus, by high fidelity of the represented environment (Dan & Reiner, 2017). Accordingly, VR environments are more pronounced regarding vividness as compared to classical setups (Slater & Wilbur, 1997), which is also characteristic for AM (Greenberg & Rubin, 2003). In particular, everyday experiences arise from the complex, multisensory 3D-environment of the real world, while laboratory memories are generated by highly controlled events rather poor in sensory information (Cabeza & St Jaques, 2007). Moreover, the formation of such memories is accompanied by intuitive and quick monitoring and closely linked to self-referential processing (Moscovitch & Winocur, 2002; Cabeza & St Jaques, 2007). Importantly, the latter is as well increased under VR conditions due to its immersive character: VR facilitates an increased sense of presence, i.e. the subjective feeling of being within a virtual environment (VE; e.g. Slater & Wilbur, 1997; Schubert et al., 2001; Nilsson, Nordahl, & Serafin, 2016). Whereas immersion predominantly determines the degree to which the user is isolated from his physical surroundings by technical factors, like 3D-360° view and proprioceptive matching, presence promotes the subjective feeling of actually being in and acting within the VE (Slater & Wilbur, 1997; Nilsson, et al., 2016). Consequently, the sensation of acting within the VE comes in with the impression of being subject to the consequences of these actions and events in the VE (Slater & Wilbur, 1997; Nilsson, et al., 2016). For example, participant behave as if being in real danger when exposed to dangerous situations in an immersive VE, even though their surroundings could not physically harm them (e.g. Kisker et al., 2019a; Krijn et al., 2004; Gromer et al., 2019). In line, VR setups have been found to elicit the same emotional and physical reactions as compared to their real-life equivalents (Gorini et al., 2010; Higuera-Trujillo et al., 2017). Given this impression of mutual interaction with the virtual surroundings, VR experiences are more personally and emotionally relevant than mere on-screen experiences (see Kisker et al., 2019a; Schöne et al., 2016, Schöne et al. 2019). Hence, VR might improve the possibilities to investigate the mechanisms underlying real-life memory (see Parsons, 2015; Serino & Repetto, 2018; Schöne et al., 2016, Schöne et al. 2019; Kisker et al., 2019b; Burgess et al., 2001).

Initial studies of memory processes under immersive VR conditions found that retrieval of VR experiences is not only enhanced compared to the retrieval of conventional laboratory micro-events (see e.g. Serino & Repetto, 2018; Smith, 2019; Schöne et al., 2016, Schöne et al. 2019; Krokos, Plaisant, & Varshney, 2019; Ernstsen, Mallam & Nazir, 2019; Harman, Joel, Brown, Ross & Johnson, 2017), but also provides a closer approximation to real-life memory processes (Schöne et al., 2016; Schöne et al. 2019; Kisker et al., 2019b). In particular, a previous study found evidence that immersive VR experiences become part of an extensive autobiographical associative network, whereas conventional video experiences remain an isolated episodic event (Schöne et al., 2019). Going one step further, the retrieval of VR experiences is proposed to mainly rely on recollection, i.e. vivid and accurate remembering of events (e.g. Atkinson & Juola, 1973; Jacoby & Dallas, 1981) which is associated with AM (Roediger & Marsh, 2003; Conway, 2005). In contrast, retrieval of memories induced by conventional laboratory settings predominantly fall back on familiarity-based mnemonic processes (Kisker et al., 2019b), characterized as a subjective, vague feeling to remember a previous experience (e.g. Curran & Hancock, 2007; Rugg & Curran, 2007). Although both groups principally employed both, familiarity and recollection as non-exclusive retrieval mechanisms (see Jones and Jacoby, 2001), one mechanism predominated over the other as a function of the encoding context. Accordingly, encoding in VR resulted in a more precise and vivid retrieval than encoding the same scenario in a PC setup (Kisker et al., 2019b).

Overall, these studies suggest that VR experiences are not just observed, i.e. passively watching stimuli presented on a screen, but experienced in a self-relevant manner. Even interactive PC setups designed as immersive as possible by means of active exploration of a desktop-based environment, generate overall rather superficial engrams compared to exactly the same VE explored as a VR experience (Kisker et al., 2019b). Unlike conventional laboratory experiences, the latter become part of a personal experience like real-life experiences would (Schöne et al., 2016, 2019).

However, while the electrophysiological correlates of, for example, the sense of presence (e.g. Bouchard et al., 2009) and spatial memory (e.g. Rauchs et al., 2008) are recently more widely investigated, findings regarding the electrophysiological correlates of retrieval of episodic and autobiographical engrams encoded within VR are still rare (cf. e.g. Smith, 2019; Serino & Repetto, 2018; Plancher & Polino, 2017; Bohil et al., 2011). Accordingly, it is the aim of our study to differentiate the electrophysiological correlates of the retrieval of VR experiences as opposed to conventional laboratory experiences. Specifically, we examined a well-established electrophysiological marker of recognition memory tasks by means of the theta old/new effect obtained from laboratory settings (for review see Nyhus & Curran 2010; Guderian & Düzel, 2005; Hsieh & Ranganath, 2014; see also Gruber, Tsivilis, Giabbiconi & Müller, 2008; Klimesch et al., 1997a, 2001a). Therefore, we examined theta-oscillations (~ 4-8 Hz; e.g. Nyhus & Curran, 2010), which are most prominent at sensors over frontal-midline regions (e.g. Hsieh & Raganath, 2014). There is broad and stable consensus, that a characteristic theta-band synchronization can be observed in these regions in response to the retrieval of old stimuli, which are correctly remembered, i.e. in response to retrieval success. In contrast, new stimuli are associated with theta-band desynchronization (e.g. Nyhus & Curran, 2010). This effect was observed both subsequent to the stimulus presentation (e.g. Klimesch et al., 1997b; Klimesch et al., 2001a) and after a physical response of participants, e.g. key pressure (Gruber, Tsivilis, Giabbiconi, & Müller, Gruber et al., 2008). Moreover, theta-oscillations are associated with recollection of personal events (Guderian & Düzel, 2005) and hippocampal projections to neocortical frontal regions are regarded as possible generators of these oscillations during memory tasks (e.g. Hsieh & Ranganath, 2014). In conjunction with the characteristic frontal-midline theta-band synchronization, a decrease of the alpha-band response (~ 8–13 Hz, e.g. Berger, 1929) can regularly be observed during memory recall (e.g. Klimesch, et al., 1997b; Sauseng et al., 2009; Jacobs, Hwang, Curran & Kahana, 2006). This decrease of alpha-band response is regarded a reflection of visual processing (Clayton, Yeung & Cohen Kadosh, 2018), attentional processes (Klimesch et al., 1997a) and memory load (Sauseng et al., 2009; Jacobs et al., 2006; Jensen & Tesche, 2002; Dan & Reiner, 2017). In short, the theta-band synchronizes in response to mental activity, whilst the alpha-band desynchronizes (Berger, 1929 as cited in Klimesch et al., Klimesch, Doppelmayr, Schimke, et al. 1997b).

To examine whether this well-established and robust effect occurs under VR conditions as well, we set up an experiment in which participants incidentally encoded either immersive 3D-360° videos or conventional 2D videos followed by an unannounced recognition memory test. We assume that the VR condition will result in a higher sense of presence, better memory performance and higher accuracy of memory judgements as compared to the conventional PC condition. Moreover, we hypothesize to replicate the theta old/new effect for the conventional PC condition, manifested significant difference between theta-band responses to old and new stimuli, including a synchronization for old, and a desynchronization for new stimuli (see e.g. Gruber et al., 2008; Klimesch et al., 1997a, 2001a, b). In line, the alpha-band response should significantly decrease for new pictures as compared to old pictures. Concerning the VR condition, different outcomes might be possible: Under the premise that the theta old/new effect is exclusively linked to successful memory retrieval, theta-band synchronization for old stimuli should be higher for the VR condition as compared to the PC condition, as most studies indicate that VR setups enhance memory performance (e.g. Schöne et al. 2016, 2019; Smith, 2019) and activate recollection-based engrams (Kisker et al., 2019b). For the alpha-band, a similar pattern of results might be expected. However, as theta-band oscillations are related to further memory-related processes, e.g. memory load (Nyhus & Curran, 2010; Jensen & Tesche, 2002), decision making (Nyhus & Curran, 2010) and working memory (Hsieh & Ranganath, 2014), another outcome than the classical effect might be equally likely in the VR condition.

## Methods

### Participants

45 participants were recruited from Osnabrück University. The sample size was determined on the basis of previous studies with a similar study design (*cf.* Schöne et al., 2019; Kisker et al., 2019a). All participants were screened for psychological and neurological disorders and had normal or corrected-to-normal sight. Three participants were excluded during the anamnesis. When vision correction was necessary, only those participants who had contact lenses could participate, not those who wore glasses. It was ensured that the participants saw sharply on the screen as well as on the head-mounted display. Previous experience with VR environments was documented. All participants gave informed consent and were blind to the research question. The participants received either partial course credits or 15€ for participation.

The participants were randomly assigned to both conditions (VR vs. PC). Three participants were excluded from analysis due to insufficient data quality (*n *= 2) and prior knowledge of the stimulus material used for the unannounced recognition memory test (*n* = 1). After exclusion, we obtained 39 complete datasets for analysis (VR group: *n*_VR_ = 20, *M*_age_=21.95, SD_age_ = 3.19, 15 female, 19 right-handed; PC group: *n*_PC_ = 19, *M*_age_=22.16, SD_age_= 2.32, 13 female, 18 right-handed).

### Encoding

#### Stimulus material

One hundred 3D-360° videos from the Library for Universal Virtual Reality Experiments (luVRe, Schöne, Kisker, Sylvester, Radtke & Gruber, 2020;  https://www.psycho.uni-osnabrueck.de/fachgebiete/allgemeine_psychologie_i/luvre.html) were used as stimulus material. All videos were recorded with the *Insta360Pro* VR-camera with a frame rate of 60 fps and 4 k resolution. Each video was 10 s long. The videos were randomly subdivided into targets and distractors for the unannounced recognition memory test in a 50:50 ratio. The themes of the videos were balanced between target and distractor videos (e.g. nature footage, interiors, medical facilities, sport events, social events; see supplementary material for a detailed description of the video content). Only the target videos were presented during incidental learning. Distractor videos were unknown to the participants and only used for the unannounced recognition memory test.

#### Procedure

Participants were randomly assigned to the VR- or the PC-condition. For the VR-condition, participants were equipped with a wireless version of the *HTC Vive Pro* head-mounted display. Video footage and sound were presented in 3D-360°, with a resolution of 1080 × 1200 pixels per display. Participants were allowed to look around, but not to turn 180° or walk around.

For the PC-condition, participants were seated in front of a curved monitor (35″, 90 cm screen diagonal, 37 cm height). The participant’s distance to the screen was kept constant at 80 cm. The videos were presented in 2D videos in full-screen resolution. Sound was presented over standard speakers placed on both sides of the monitor.

**Fig. 1:**
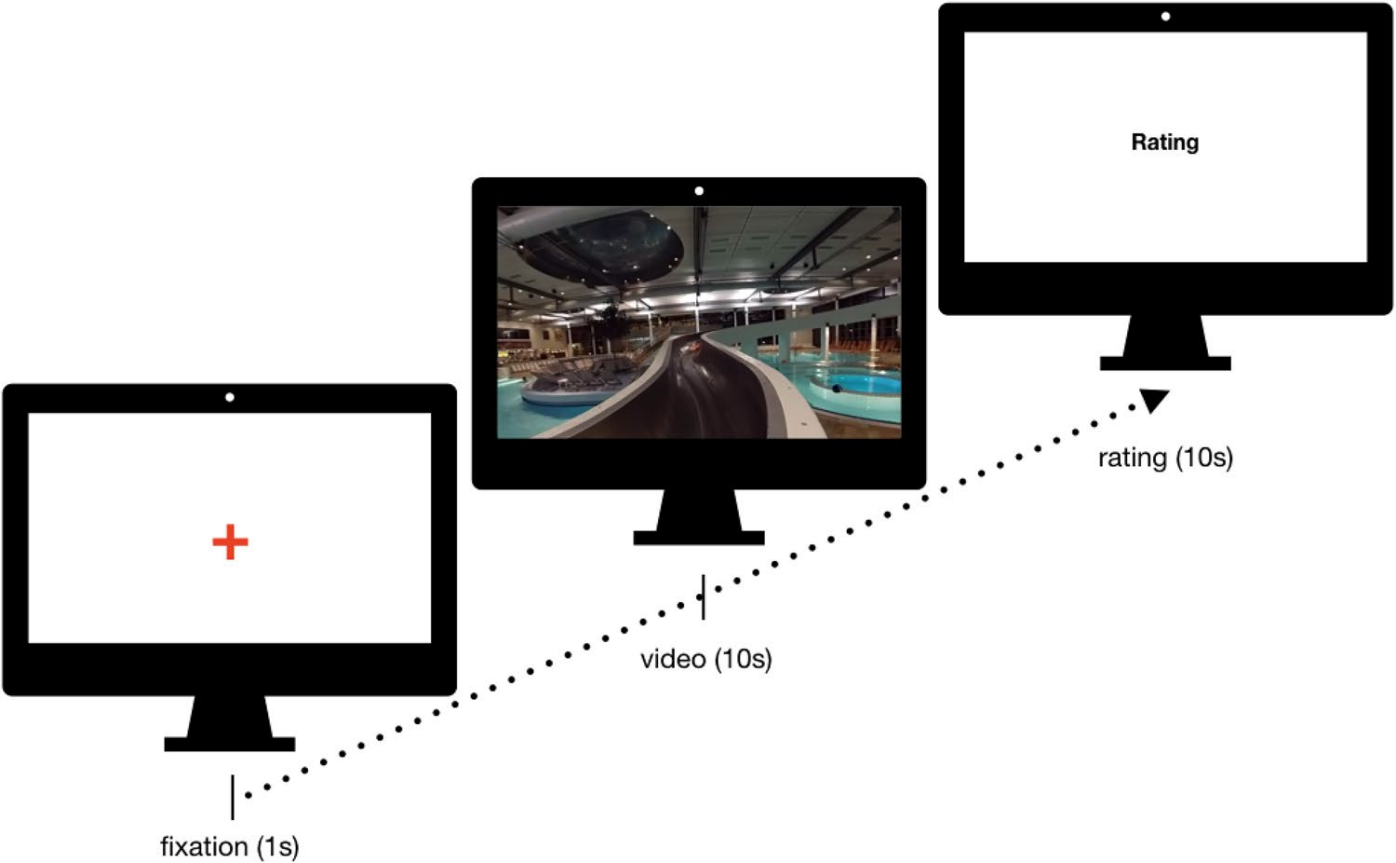
Procedure of incidental encoding. Each of the 50 target videos was preceded by a fixation on a virtual screen and followed by the rating of valence, arousal and motivation. Each scale was faded in on the virtual screen separately for 3.33 s. During fixation and rating, a 3D-360° image of the laboratory in which the participants were actually located was presented

For both conditions, the videos were presented in randomized sequences with the *GoPro VR Player,* providing the same video resolution for both conditions (cf. stimuli). Each randomized sequence was presented to one participant per condition. Each video was preceded by one-second fixation on a fixation cross. To facilitate incidental encoding, the presentation of each video clip was followed by a rating (10 s) as a distraction task (cf. Fig. 1). Participants were instructed to separately rate the experienced valence, arousal and motivation, i.e. their desire to stay in or leave the presented scene for each video separately on a scale from one (bad/not at all) to six (good/very much; cf. Kuhr et al., 2015). The ratings were consecutively presented on the (virtual) screen for 3.33 s each. The participants were familiarized with the rating before the video presentation. To guarantee for similar visual experience during the rating and maintain immersion, the rating took place in an exact virtual simulation of the laboratory in which the study actually took place, implemented as a 3D-360° video recording of the laboratory. In addition, the rating scales were displayed on the (virtual) monitor during rating phase. For the PC group, the simulation of the laboratory was displayed as a 2D video as well. The participant’s answers were recorded with a dictation device. The ratings regarding valence, arousal and motivation of the videos were collected for the validation of a database and will not be further analyzed in this study. The presentation of the videos took a total of 19 min. To enhance immersion, all test leaders left the lab until the end of the video presentation. The participant was given a bell to alert the test leaders if they wanted to quit the experiment early or felt uncomfortable.

To determine the sense of presence, participants were asked to fill in the German version of the *Igroup Presence Questionnaire* (*IPQ*; Schubert, Friedmann & Regenbrecht, 2001) and were asked for their experience of physical symptoms (vertigo, nausea). In addition, the participants were instructed not to discuss the videos with the test leaders until the end of the experiment.

### Unannounced recognition memory test

#### Stimulus material

Monoscopic screenshots from both, distractors (referred to as new pictures) and targets (referred to as old pictures), were used as stimulus material for the unannounced recognition memory test. Per video, one representative screenshot was utilized as stimulus, resulting in 100 trials. The stimuli were presented on a conventional 24″ monitor with a parafoveal visual angle of 2 × 5°.

#### Procedure

The retention interval was set to 1 h during which the EEG was applied. If the participants mentioned the videos they had seen during encoding, they were kindly interrupted and asked not to discuss the videos until the end of the experiment. Participants were instructed about their task immediately before the unannounced recognition memory test.

**Fig. 2:**
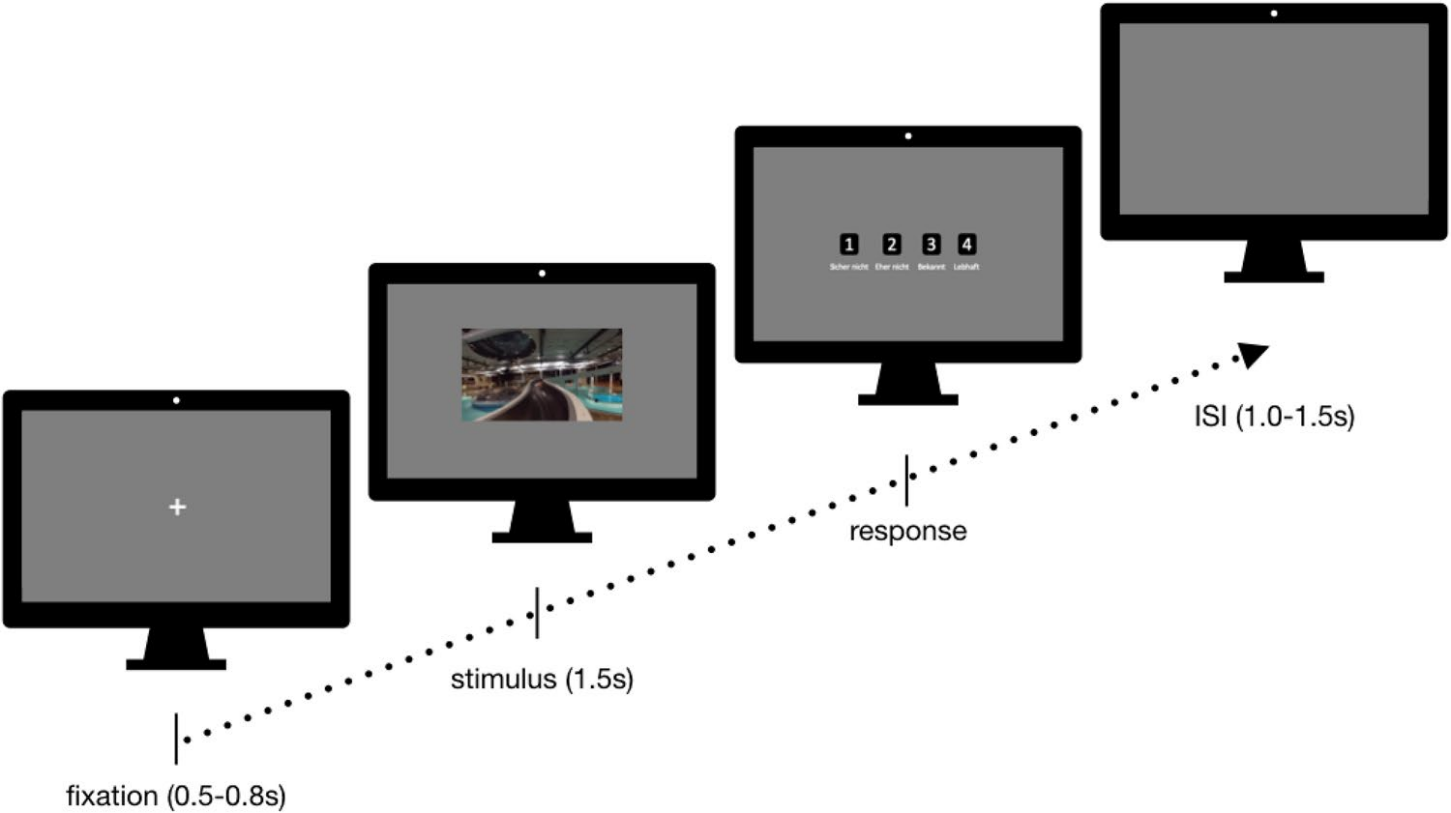
Setup of the memory test trials: 0.5–0.8 s fixation, 1.5 s stimulus presentation, presentation of the scale until the participant’s response, 1.0–1.5 s inter stimulus interval (ISI). Participants were asked not to blink from fixation until the response scale appeared

The unannounced recognition test comprised of 100 trials. Per trial, participants had to indicate as fast as possible whether they recognized the presented stimulus as (1) definitely unknown, (2) rather unknown, (3) familiar or (4) vividly remembered (cf. Kisker et al., 2019b). Each trial started with randomly 0.5–0.8 s fixation, followed by 1.5 s presentation of the stimulus. The rating scale was then displayed until the participants responded via key pressure. The interstimulus interval lasted randomly between 1.0 s and 1.5 s (see Fig. 2). The response options were defined during instruction as follows (translated from German):

1. Definitely unknown: I’m sure I’ve not seen this place
2. Rather unknown: I guess I haven’t seen this place
3. Familiar: This place looks familiar to me
4. Vividly remembered: I remember this place precisely and vividly.

#### Electrophysiological recordings and preprocessing

An electroencephalogram (EEG) with 128 electrodes, attached in accordance with the international 10-20-system was recorded for the duration of the unannounced recognition memory test. The Active-Two amplifier system from BioSemi (Amsterdam, Netherlands) was used. The sampling rate was 1024 Hz, the bandwidth (3 dB) 104 Hz. Additionally, horizontal electrooculogram (hEOG) and vertical electrooculogram (vEOG) were recorded and a common mode sense (CMS) and a driven right leg (DRL) electrode were applied. The EEG was recorded on the investigators’ computer using ActiView702 Lores.

EEG data were analyzed using MATLAB. For further off-line analysis, the average reference was used. The EEG was segmented to obtain epochs starting 500 ms prior and 1500 ms following stimulus onset (baseline − 300 to − 100 ms). Artifact correction was performed by means of ‘‘statistical correction of artifacts in dense array studies’’ (SCADS; Junghöfer, Elbert, Tucker, & Rockstroh, 2000). In brief, this procedure uses a combination of trial rejection and channel approximation based on statistical parameters of the data. For each trial, contaminated electrodes are detected based on a threshold criterion derived from the distribution of the amplitude, standard deviation, and gradient of the sensor across all trials. The information of these electrodes is replaced with a spherical interpolation from the full channel set. The limit for the number of approximated channels was set to 20. Epochs containing more than 20 channels with artifacts were rejected.

For demonstrating a robust signal at the frequency bands of interest, we first calculated a conventional fast Fourier transform (FFT, see Fig. 5) per trial and averaged across all electrodes, conditions and participants.

For further analyses and a comparison between experimental conditions, we considered it advantageous to take the signal’s temporal evolution into account. Thus, for the subsequent examinations, spectral changes in oscillatory activity were analysed by means of Morlet wavelets with a width of 12 cycles per wavelet which is described in detail elsewhere (e.g., Tallon-Baudry & Bertrand, 1999; Bertrand & Pantev, 1994). In brief, the method provides a time-varying magnitude of the signal in each frequency band, leading to a time-by-frequency (TF) representation of the data. Due to the fact that induced oscillatory activity occurs with a jitter in latency from one trial to another (Eckhorn et al., 1990), they tend to cancel out in the averaged evoked potential. Thus, TF amplitude is averaged across single-trial frequency transformations, allowing one to analyze non-phase-locked components. Furthermore, because we focused on the non-phase-locked components of the signal, the evoked response (i.e., the ERP) was subtracted from each trial before frequency decomposition (for details, see Busch, Herrmann, Müller, Lenz, & Gruber, 2006). Given our interest in the lower-frequency range, we used wavelets from 0.25 Hz to 30 Hz.

Based upon prior literature (e.g. Nyhus & Curran, 2010) and our hypothesis, the frequency range from 4-7 Hz was included in the analyses and checked against visual inspection of the FFT (see Fig. 5). However, visual inspection of the FFT revealed high power for 2–4 Hz as well. This frequency range is commonly denoted as the delta-band, but was also identified as lower theta-band in some studies, indicating that the old-new effect might be reflected in the 2–4 Hz frequency range as well (cf. Burgess & Gruzelier, 1997; Klimesch, Schimke & Schwaiger, 1994; Klimesch et al., 2000). Hence, the 2–4 Hz response was included in the analyses as well. Electrodes around *Fz* covering for the frontal midline region were chosen. Based upon prior literature, an early latency range from 250 to 650 ms for the 2–4 Hz response (see Burgess & Gruzelier, 1997) and 200–600 ms for the 4–7 Hz band response were used for analyses (e.g. Guderian & Düzel, 2005; Klimesch et al., 1997b; Klimesch, Doppelmayr, Schwaiger, Winkler & Gruber, 2000; Klimesch al., 2001b; Jacobs et al., 2006). The alpha frequency band (8–13 Hz, see e.g. Berger, 1929) was analyzed at electrodes surrounding Oz, O1 and O2 in the time window from 0 to 500 ms.

### Statistical analysis

#### Presence

The IPQ scales were determined as sum values of the respective items (in total: 14 items; general presence: one item, spatial presence: five items, involvement: four items, realness: four items). Each item could reach values from − 3 and + 3 on a 7-step likert-scale, resulting in the following minimum and maximum sumscores per scale: *General Presence* (− 3; 3), *Spatial Presence* (− 15; 15), *Involvement* (− 12; 12), *Realness* (− 12; 12).

Shapiro–Wilk-test rejected normal distribution for one of the *IPQ* scales (*General Presence*, *p* < 0.05). Therefore, the more robust Mann–Whitney *U* test as non-parametric equivalent of the unpaired *t* test was used for analysis. *Cronbach’s α* was calculated for each scale, with the exception of the one-item-scale *General Presence.*

#### Memory performance

*D*′-prime (*d*′) was calculated separately for both groups as an operationalization of memory performance. *D’* relates the hits, i.e. correct positive judgments, to the false-positive judgments (*d’ *= *z*(hit) −− *z*(false positive); Haatveit et al. 2010; Swets et al., 1961; as cited in Kisker et al., 2019b) and indicates how well participants are able to distinguish between targets and distractors. *D’*-prime was calculated per group to assess the overall retrieval success (general *d’ *=* z*(all hits) – *z*(all false positives)).

Additionally, *d’* was separately calculated for familiarity and recollection for each group, taking only the respective hits and false positives into account (cf. Kisker et al., 2019a: *d’*-familiarity score = *z*(familiarity hits) – *z*(familiarity false positives); *d’*-recollection score = *z*(recollection hits) – *z*(recollection false positives). Shapiro–Wilk-test rejected normal distribution for all *d’* scores (all *p* < 0.05). Hence, Mann–Whitney *U* Test was used for analysis.

Accuracy [(hits + correct rejection)/total number of trials] and error rate [(misses + false positives)/total number of trials] of recognition judgements were calculated per group. Both were analyzed using the unpaired *t* test.

#### Prior VR experience and cybersickness

Prior experience with VR and cybersickness were assessed as nominal variables (prior experience: “*Have you already had any experience with virtual reality, e.g. studies, games or videos*?”, [yes/no]; cybersickness: “*Did you experience physical symptoms such as nausea or dizziness during the experiment?*”, [yes/no]; if yes: “*How strongly did you feel nauseous/dizzy*?” [1–10]; cf. Kisker et al., 2019a). Contingency tables and Pearson’s Chi square (*X*^*2*^) test were used for statistical analysis.

#### Ratings of the videos

The ratings regarding valence, arousal and motivation of the videos were collected for the validation of a database and will not be further analyzed in this study. To check that the target videos were perceived comparably emotive in both groups, arousal and valence averaged over all 50 target videos were compared between the groups using unpaired* t* test.

#### Dependent measures

EEG data were analyzed using a 2 × 2 *repeated*-*measurements ANOVA* (rmANOVA) with the between-factor “group” (VR vs. PC) and the within-factor “condition” (new pictures vs. old pictures). Significant effects of *rmANOVA* were complemented by post hoc *t* tests.

## Results

### Subjective measures

#### Presence

**Fig. 3:**
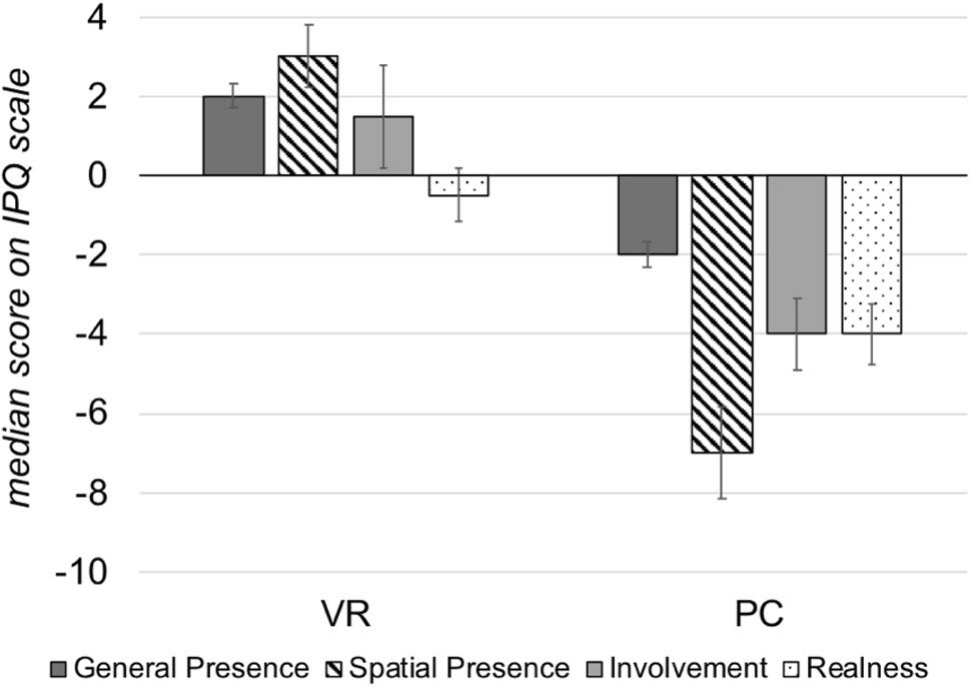
Median scores of the IPQ scales General Presence, Spatial Presence, Involvement and Realness as evaluated by both groups. The error bars depict the standard error per scale. Minimum and maximum sumscores per scale: General Presence (− 3; 3), Spatial Presence(− 15; 15), Involvement (− 12; 12), Realness (− 12; 12)

As hypothesized, the VR-group reported a higher feeling of presence during video presentation (see Fig. 3). This is valid for all *IPQ* subscales (all *p* ≤ 0.005; see Table 1). Cronbach’s *α* indicates acceptable reliability for all scales (all *α* ≥ 0.64).

**Table 1:** Differences between VR- and PC-group regarding the sensation of presence, assessed via the IPQ (Schubert et al., 2001): Test statistics of the one-tailed Mann–Whitney *U* test, descriptive values and Cronbach’s α per scale. Cronbach’s α could not be calculated for the one-item-scale General Presence

IPQ scale	*U*	*z*	*p*	*Md*_VR_	*Md*_PC_	Cronbach’s *α*
General presence	41.00	− 4.29	< .001	2.00	− 2.00	
Spatial PRESENCE	19.00	− 4.72	< .001	3.00	− 7.00	0.68
Involvement	98.00	− 2.59	0.005	1.50	− 4.00	0.70
Realness	64.50	− 3.55	< .001	-0.50	− 4.00	0.64

#### Prior VR experience and cybersickness

In both groups, about 70% of the participants had already gained experience with VR prior to the study, e.g. by participating in other studies, watching VR videos or playing VR-games (*X*^*2*^(1) = 0.011, *p* = 0.915). In total, nine subjects (*n*_VR_: six, *n*_PC_: three) reported experiencing physical symptoms like nausea and dizziness, but on a very mild level (nausea, in total: *M* = 2.55, SD = 2.13; VR: *M*_VR_ = 3.33, SD_VR_ = 2.25; PC: *M*_PC_ = 1.0, SD_PC_ = 0.0; dizziness, in total: *M* = 1.67, SD = 1.12; VR: *M*_VR_ = 2.00, SD_VR_ = 1.27; PC: *M*_PC_ = 1.0, SD_PC_ = 0.0), resulting in significantly stronger experiences of physical symptoms in the VR condition (*X*^*2*^(1) = 4.91, *p* = 0.027).

#### Ratings of the videos

Participants of both groups reported equal levels of valence and arousal averaged across all target videos (valence: *M*_VR_ = 3.89, SD_VR_ = 0.52, *M*_PC_ = 3.61, SD_PC_ = 0.47, *t*(34) = 1.67, *p* = 0.103; arousal: *M*_VR_ = 2.64, SD_VR_ = 0.55, *M*_PC_ = 2.65, SD_PC_ = 0.47, *t*(34) = − 0.28, *p* = 0.978).

### Memory performance

**Fig. 4:**
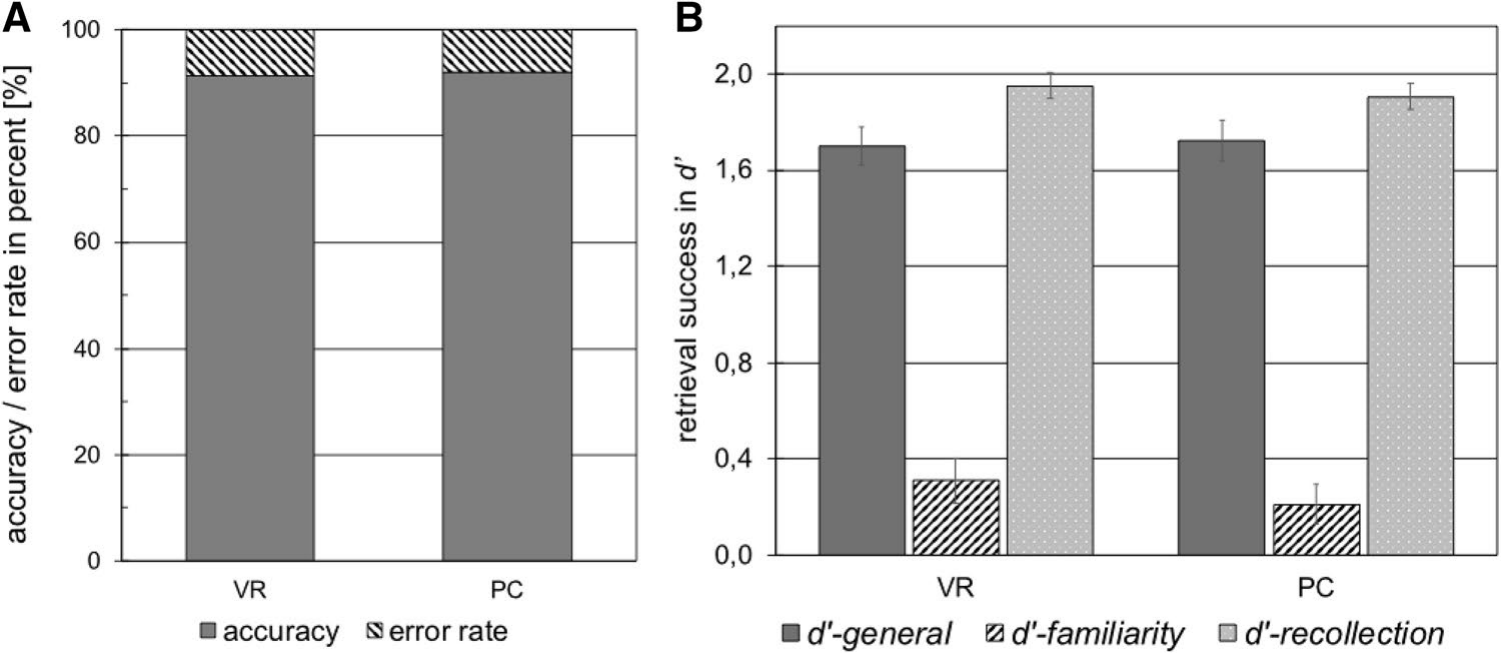
Panel A depicts the accuracy as well as the respective error rate of the judgement on the recognition or unknown character of the memory task trials in percent for both groups. The error bars depict the standard errors. For accuracy and error rate, the standard error is approximately 0.01 and therefore hardly visible in the figure. Panel B depicts the retrieval success per group operationalized by general d’ prime, as well as the d’-familiarity and d’-recollection scores. No significant differences were found between both groups

Participants of both groups performed equally well on the unannounced recognition memory test, as none of the calculated *d*′ scores revealed significant differences (*d’*-general: *U* = 186.00, *z* = − 0.11, *p* = 0.462; *d*′-familiarity: *U* = 150.50, *z* = − 1.11, *p* = 0.14; *d’*-recollection: *U* = 162.50, *z* = − 0.77, *p* = 0.22; see Fig. 4).

Moreover, both groups achieved surprisingly high levels of accuracy around 90% (*t*(37) = − 0.505, *p* = 0.308, *M*_VR_ = 0.91, *M*_PC_ = 0.92) and correspondingly low error rates (*t*(37) = − 0.505, *p* = 0.31, *M*_VR_ = 0.09, *M*_PC_= 0.08; see Fig. 4), indicating a ceiling effect.

### Dependent measures

Since the behavioral data indicate no difference in memory performance between both groups, and since the high accuracy indicates a ceiling effect, the latency range following stimulus onset was analyzed instead of the latency range following the participants’ response (key pressure) to the stimulus (cf. results, memory performance).

**Fig. 5:**
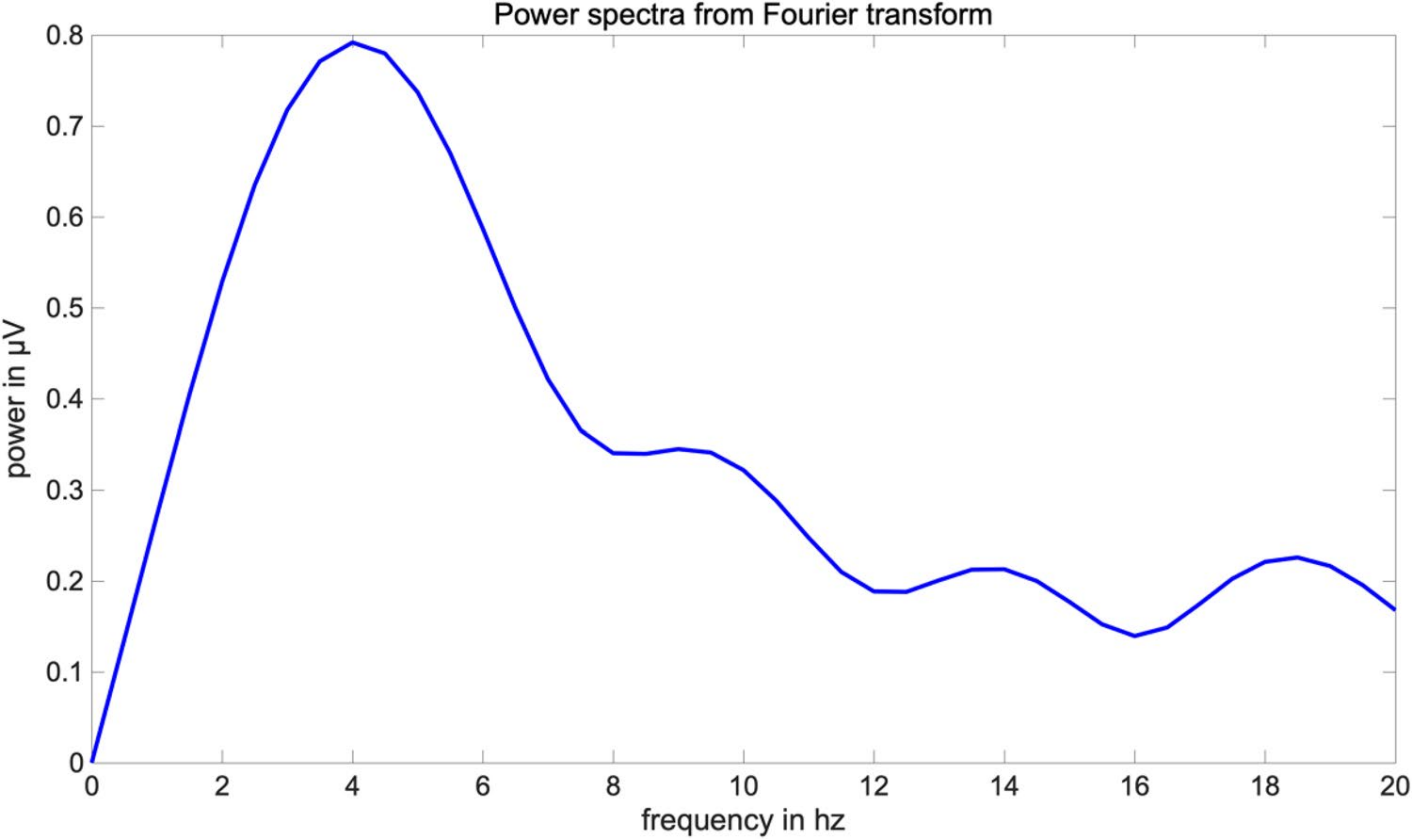
Power spectra from fast Fourier transform (FFT) per group and condition. Visual inspection revealed a strong frequency peak from 2 to 4 Hz, which was hence included in the analyses

The visual inspection of the FFT validated the hypothesis-driven selection of the 4–7 Hz and 8–13 Hz frequency ranges. In addition, the visual inspection also revealed a noticeable power of the 2–4 Hz frequency range, which is why it was also included in the analyses (see Fig. 5, see methods).

#### 4–7 Hz responses

Regarding frontal-midline theta-band responses, no significant main effects could be found (*F*_condition_(1,37) = 2.84, *p* = 0.10; *F*_group_(1,37) = 0.38, *p* = 0.543), but a significant interaction of the factors “group” and “condition” (*F*_interaction_(1,37) = 5.03, *p* = 0.046).

**Fig. 6:**
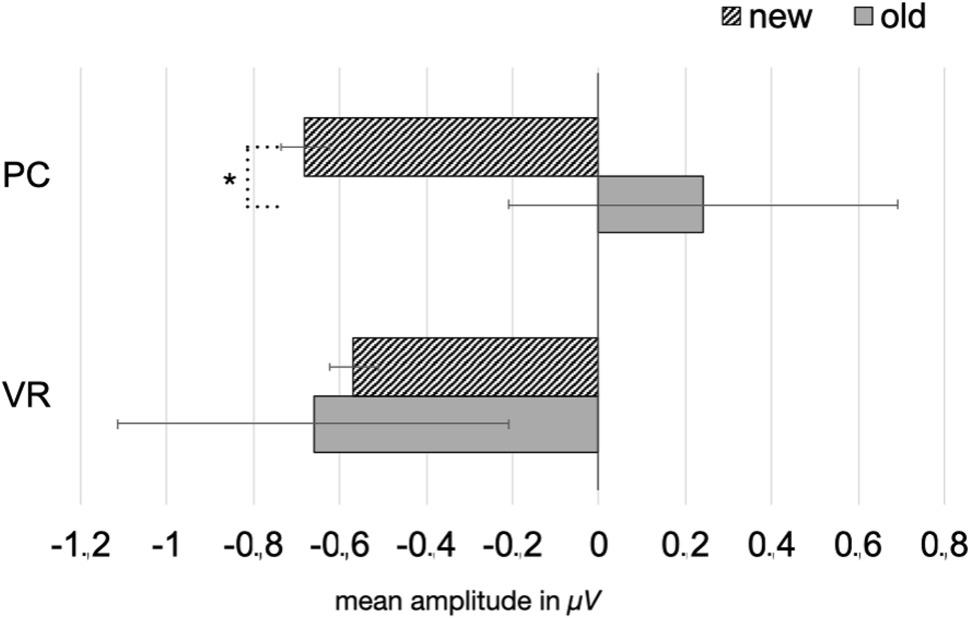
Mean amplitude in µV regarding the 4–7 Hz response in the latency range from 200 to 600 ms after stimulus onset. The error bars depict the standard error of the mean amplitude. Significant differences are marked (*p < 0.05)

**Fig. 7:**
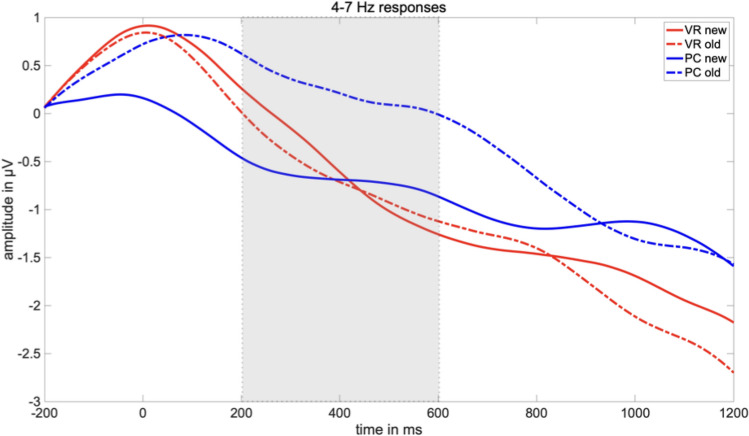
Time-by-amplitude plot of the 4–7 Hz response from 200 ms before stimulus onset to 1200 ms after stimulus onset. While the classical old/new-effect is also descriptively shown in the PC condition, there are no significant differences between old and new pictures regarding the VR-group. The gray highlighted section  marks the latency range of significant interaction. The amplitude was averaged across the electrodes around Fz, covering for the frontal midline region

**Fig. 8:**
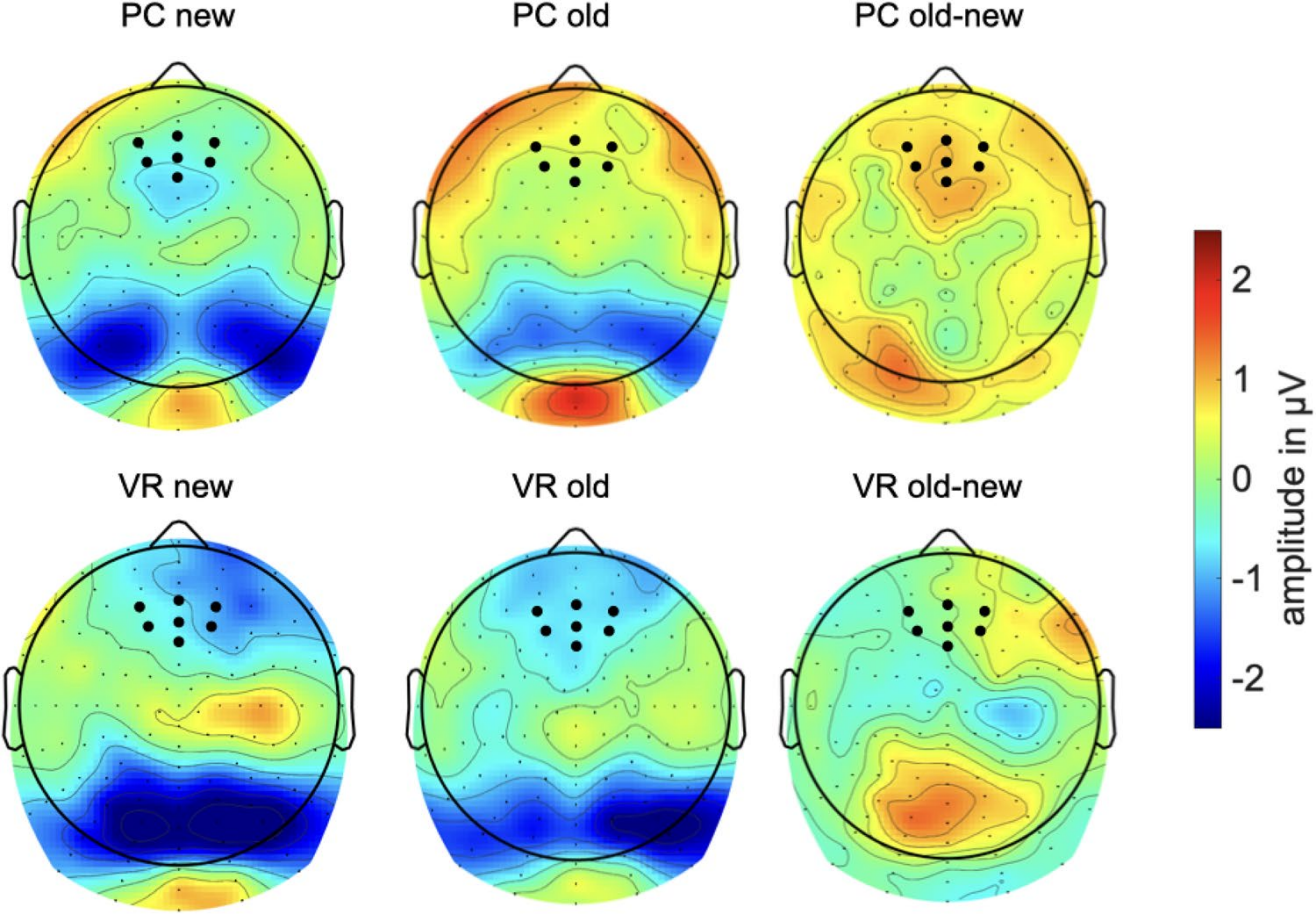
Topography of the amplitude regarding the 4–7 Hz response separately for all combinations of the factors group (VR vs. PC) and conditions (old vs. new) in the latency range from 200 to 600 ms after stimulus onset. Additionally, a difference plot of the old/new-effect is depicted. Black dots mark the electrodes which were included in the analyses

Post-hoc *t* tests revealed a classical old/new effect in the PC condition with a higher amplitude for old pictures than for new ones (*t*(18) = − 2.86, *p* = 0.010). However, this difference effect was absent within the VR condition (*t*(19) = 0.25, *p* = 0.805). Furthermore, the observed difference effect was comparably larger in the PC-group (*t*(37) = 2.06, *p* = 0.046; see Figs. 6 and 7). The theta-band response to new pictures (*t*(37) = − 0.14, *p* = 0.889) and to old pictures (*t*(37) = 1.65, *p* = 0.107) did not differ between both groups (see Figs. 6, 7 and 8).

#### 2–4 Hz responses

**Fig. 9:**
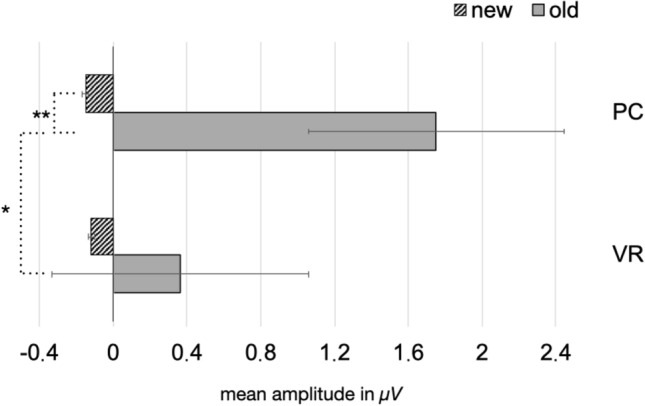
Mean amplitude in µV regarding the 2–4 Hz response in the latency range from 250 to 650 ms after stimulus onset. The error bars depict the standard error of the mean amplitude. Significant differences are marked (*p < 0.05; ** *p* < 0.01)

**Fig. 10:**
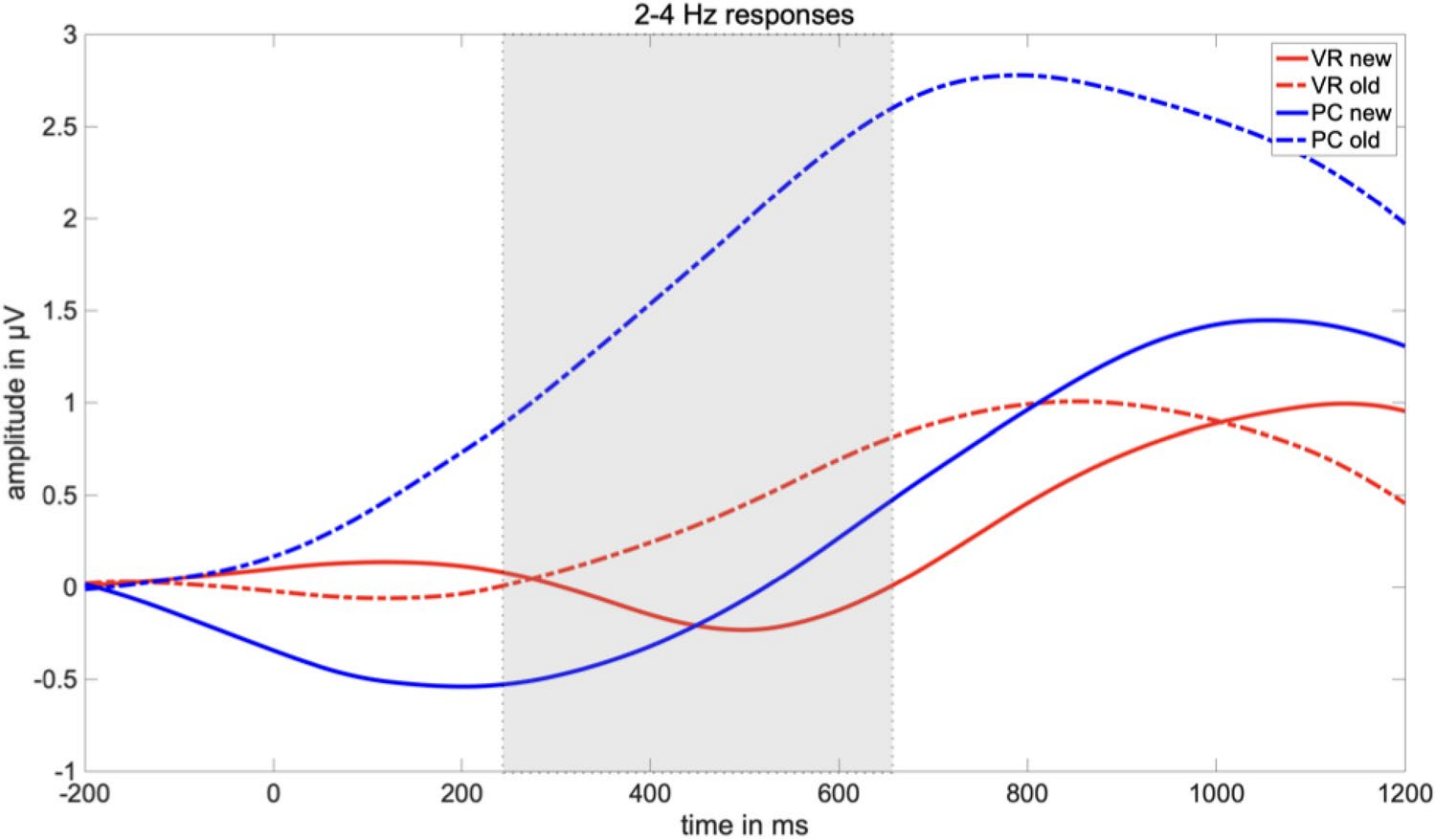
Time-by-amplitude plot of the 2–4 Hz response from 200 ms before stimulus onset to 1200 ms after stimulus onset. The gray highlighted section  marks the latency range of significant interaction. The amplitude was averaged across the electrodes around Fz, covering for the frontal midline region

**Fig. 11:**
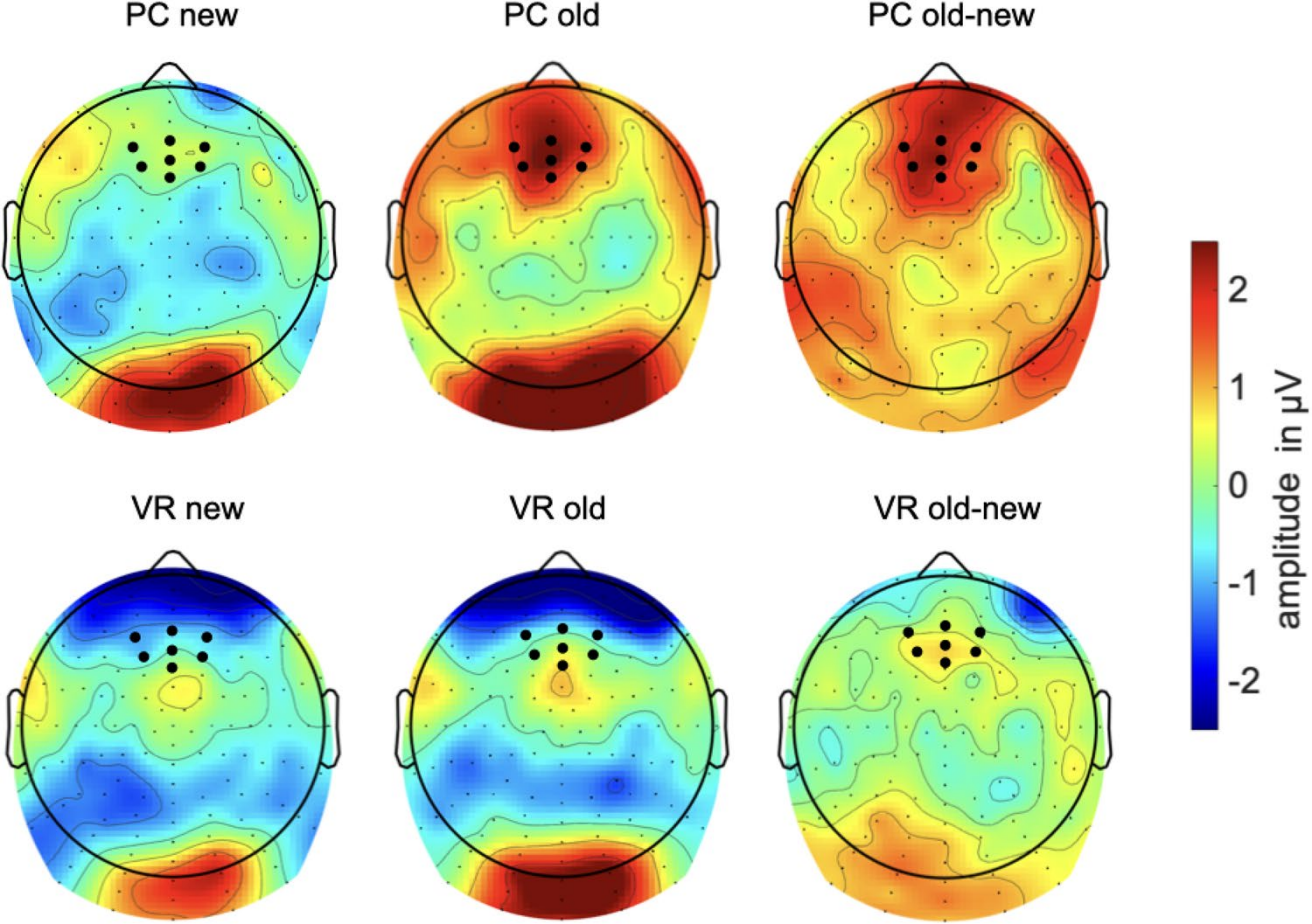
Topography of the amplitude regarding the 2–4 Hz response separately for all combinations of the factors group (VR vs. PC) and conditions (old vs. new) in the latency range from 250 to 650 ms after stimulus onset. Additionally, a difference plot of the old/new-effect is depicted. Black dots mark the electrodes which were included in the analyses

For the 2–4 Hz response, a significant main effect for the factor “condition” (*F*_condition_(1,37) = 11.61, *p* = 0.002), but not for the factor “group” (*F*_group_(1,37) = 1.44, *p* = 0.239) could be found. The main effect of “condition” was further characterized by a significant interaction of both factors (*F*_interaction_(1,37) = 4.11, *p* = 0.049). Following the same trend as the 4-7 Hz responses, post hoc *t* tests revealed a classical old/new effect across conditions (*t*(38) = 3.23, *p* = 0.003), as well as in the PC condition (*t*(18) = − 4.74, *p* < 0.001), but not in the VR condition (*t*(19) = − 0.85 *p* = 0.404). Again, the observed difference effect was comparably larger in the PC-group (*t*(37) = 2.03, *p* = 0.049). But most importantly, old pictures elicited greater responses in the PC group compared to the VR-group (*t*(37) = 2.07, *p* = 0.046), whereas responses to new pictures did not differ between both groups (*t*(37) = − 0.05, *p* = 0.96; see Figs. 9, 10 and 11).

#### Alpha-band responses

Regarding the alpha-band responses (8–13 Hz) at occipital electrodes, a main effect of the factors group (*F*_group_(1,37) = 4.26, *p* = 0.046) and condition (*F*_condition_(1,37) = 13.80, *p* < 0.001), but no significant interaction of both factors was found (*F*_interaction_(1,37) = 1.21, *p* = 0.278).

**Fig. 12:**
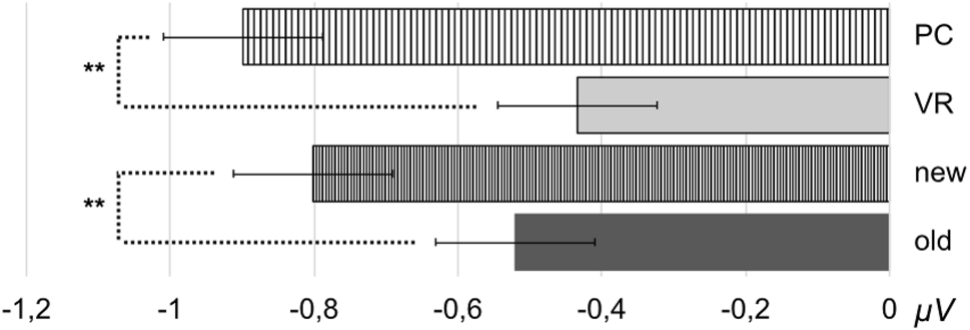
Mean alpha amplitude (8-13 Hz) in µV in the latency range from 0 to 500 ms after stimulus onset. The error bars depict the standard error of the mean amplitude. Significant differences are marked (*p < 0.05; **p < 0.01)

**Fig. 13:**
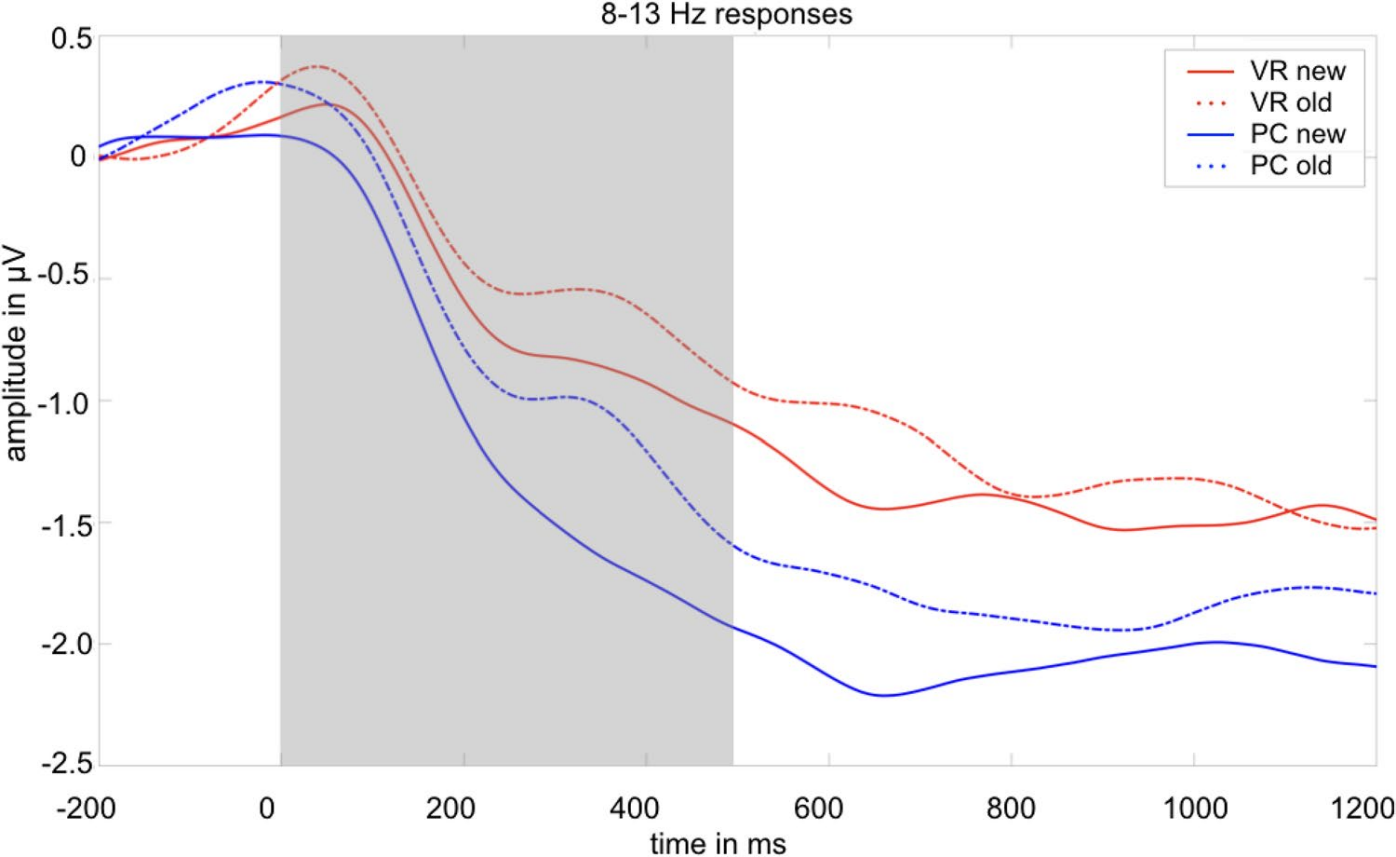
Time-by-amplitude plot of the alpha-band response (8–13 Hz) from 200 ms before stimulus onset to 1200 ms after stimulus onset. Descriptively, a stronger reduction of the alpha amplitude was observed for both PC conditions compared to both VR conditions. The gray highlighted section  marks the latency range of both significant main effects

**Fig. 14:**
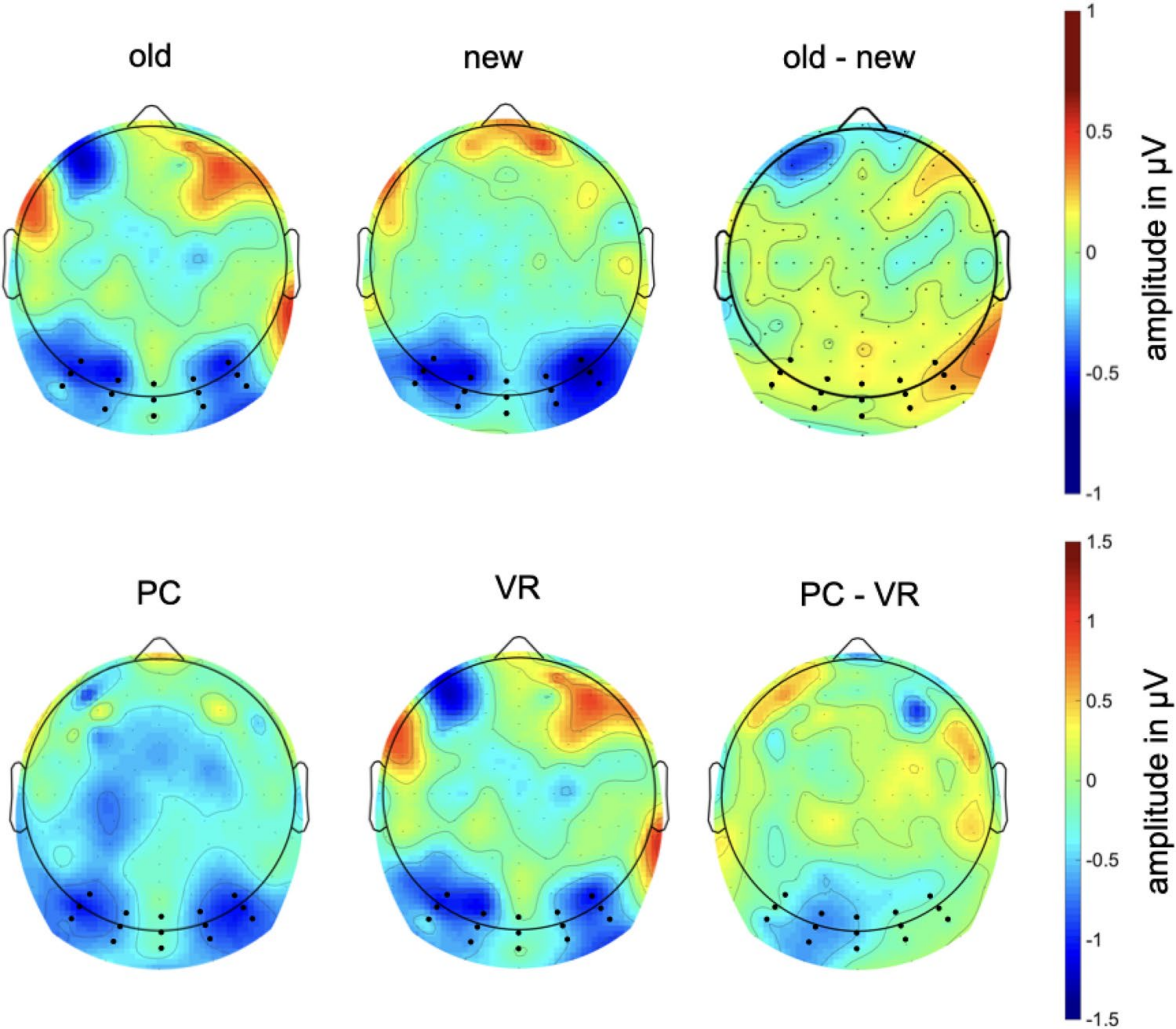
Topography plot of alpha amplitude in µV (8–13 Hz) separately for factors group (VR vs. PC) and conditions (old vs. new) in the latency range from 0 to 500 ms after stimulus onset. Additionally, a difference plot of old minus new and PC minus VR is depicted. Black dots mark the electrodes which were included in the averaged amplitude

More specifically, new pictures elicited lower alpha amplitudes as compared to old pictures (*t*(38) = 3.68, *p* < 0.001). In line, alpha amplitudes were significantly lower for the PC group as compared to the VR group (*t*(76) = 2.75, *p* = 0.008; see Figs. 12, 13 and 14).

## Discussion

The aim of the study was to investigate the electrophysiological correlates of the retrieval of VR experiences as opposed to conventional laboratory experiences. To this end, participants watched either 3D-360° VR videos (VR condition) from the *luVRe* database (see methods), or watched the exact same stimulus material on a conventional 2D monitor (PC condition). In an unannounced recognition test, we compared their memory performance, the mid-frontal theta old/new effect indexing mnemonic processing, as well as posterior alpha as a marker for visual processing load. As a result, both groups performed equally well in the recognition test, although the theta old/new effect could only be replicated for the PC condition and was absent in the VR condition. Additionally, the theta effect was accompanied by a profound reduction of posterior alpha in the PC condition, indicating a visually guided, effortful retrieval process.

Meeting our expectations, participants of the VR condition felt more present during video presentation as compared to the PC condition, confirming that our video approach led to immersive VR experiences. Presence, as the most prominent feature of VR experiences (e.g. Schubert et al., 2001, Pan & Hamilton, 2018; Diemer et al., 2015; Alshaer, Regenbrecht, & O’Hare, 2017; Riva et al., 2007; Kisker et al., 2019a), is associated with increased emotional involvement (e.g. Gorini et al., 2010; Felnhofer et al., 2015), and stronger and more realistic behavioral responses as compared to conventional laboratory settings (Slobounov et al., 2015; Kisker et al., 2019a). Importantly, previous studies found that a high degree of presence aids memory recall: For example, both intentional encoding, as well as incidental encoding in a VE resulted in a more accurate memory recall as compared to conventional desktop conditions (e.g. Krokos, Plaisant & Varshney, 2019; Ernstsen, Mallam & Nazir, 2019). Hence, presence might facilitate encoding processes constituting the VR memory superiority effect (Makowski, Sperduti, Nicolas & Piolino, 2017; Serino & Repetto, 2018; Smith, 2019). In particular, visually detailed environments that provide high realism and resemblance to the real world, such as 3D-360° videos (Pan & Hamilton, 2018; Lovett et al., 2015), facilitate more accurate judgments in old/new tasks (Smith, 2019). The resulting coherent egocentric perspective facilitates recollection and reliving of such content (see Rubin & Umanath, 2015), which is crucial to form vivid, real-life memories (Conway, 2005; Roediger & Marsh, 2003). Hence, a high sense of presence—including sensations of spatial presence, involvement and realness—means that these events are potentially significant for the participant, consciously experienced and thus, might contribute to the formation of autobiographical memory.

However, at odds with previous research (Schöne et al., 2019; Smith, 2019; Kisker et al., 2019b), our study did not provide any behavioral evidence for this effect: Even though the VR group reported higher sensations of presence as compared to the PC group, we did not observe superior memory recall performance. Our results, with both groups having an accuracy of ca. 90%, indicate a ceiling effect, limiting the detection of group differences (Bortz & Döring, 2005). A possible cause of is effect might be the short retention interval between encoding and retrieval. Previous studies, which did not apply EEG measurements, chose longer retention intervals that included one or two sleeping periods (Schöne et al., 2019; Kisker et al., 2019b). It is possible that the process of forgetting irrelevant information had not yet started at the time of the EEG measurement or had at least not progressed very far (cf. Wang, Subagdja, Tan & Starzyk, 2012). However, other studies have not been able to demonstrate this overall memory superiority of VR experiences either (LaFortune & Macuga, 2018; Dehn et al., 2018; Kisker et al., 2019b). Differences regarding the findings of VR studies might be related to varying implementations of VR technology, ranging from highly immersive head-mounted displays and CAVE systems to less immersive desktop-VR implementations (Smith, 2019). Additionally, the level of multi-sensory sensations provided by the VR system might influence memory performance as well: For example, active navigation through a VR environment can have an additional positive effect on spatial memory, but not necessarily on factual memory (Plancher, Barra, Orriols & Piolino, 2013). Moreover, some studies report a successful transfer of content learned in an immersive VR environment to real-life, and thus, to other than the encoding context (Ragan, Sowndararajan, Kopek & Bowman, 2010; as cited in Smith, 2019), whereas other studies claim that knowledge transfer comes with a loss of performance (Lanen & Lamers, 2018).

Even though VR experiences do not necessarily increase the retrieval success as measured by subjective reports, the immersive nature of VR yet might alter the mode of operation of the mnemonic mechanisms. Specifically, Kisker et al., (2019a) demonstrated by means of a remember/know paradigm that participants who explored a virtual village in an immersive VR condition report predominantly recollection-based memory. Interestingly, recollection is hypothesized to be the associated retrieval mechanism of autobiographical memory (Roediger & Marsh, 2003; Conway, 2005). Participants exploring the very same village in a PC condition reported predominantly familiarity-based memories (Kisker et al., 2019a). However, both groups in our experiment apparently employed the same retrieval strategies as the *d′*-scores for recollection, familiarity and overall performance do not differ significantly.

Nevertheless, modulations of the frontal-midline theta effect might still indicate the involvement of different types of memory systems as well as associated encoding and retrieval strategies with respect to the encoding condition. As expected, we replicated the frontal-midline theta old/new effect in the PC condition: Old pictures evoked an early theta-band synchronization, whereas new pictures resulted in theta-band desynchronization. Hence, our findings replicate broad and stable evidence relating relatively higher theta-band amplitudes to the retrieval of old, and relatively lower amplitudes to the retrieval of new pictures in conventional laboratory settings (e.g. Gruber et al., 2008; Klimesch et al., 1997a, b, 2001a, b). The change of modality, i.e. encoding videos, but retrieval in response to picture presentation, did not markedly affect the theta old/new effect in the PC condition.

Remarkably, the theta old/new could not be observed in the VR condition. Specifically, new pictures led to the same theta-band response in both groups, indicating that the physical discrepancies between encoding in VR or under conventional conditions did not affect the paradigm per se or at least affected it to the same extent. Moreover, memory success did not account for the different electrophysiological responses as well, as both groups performed equally well in the recognition test. Accordingly, differences in the electrophysiological response must result from different underlying retrieval mechanisms and thus, differences in mnemonic processing of engrams encoded from either VR experiences or conventional laboratory events. Evidence that the absence of the theta old/new effect under VR conditions results from an altered mnemonic processing style as compared to the PC condition is obtained from the comparison of the response to old pictures between both groups. Regarding the 2–4 Hz frequency range, the presentation of old pictures led to a significant difference between relative synchronization in the PC group and in the VR group. Descriptively, the 4–7 Hz frequency range follows the same trend but did not reach significance. Hence, the theta old/new effect is modulated by the nature of the engram resulting from VR experiences and how these experiences are recalled.

As aforementioned, immersive VR experiences are considered to facilitate the formation of autobiographical memory. Associative autobiographical engrams are generated by highly self-relevant experiences (Roediger & Marsh, 2003; Conway, 2005). They are characterized by richer content and are deeply interwoven into existing memory structures (McDermott et al., 2009; Roediger & Marsh, 2003). Furthermore, they come with a broad set of functional properties, namely self-reflection, emotional evaluation and semantic processes (Svoboda et al. 2006). Frontal-midline theta has repeatedly been shown to reflect key-elements of autobiographical mnemonic processing. Specifically, it is associated with the recollection of personal events and contextual information (Guderian & Düzel, 2005; Hsieh & Ranganath, 2014; see also Roediger & Marsh, 2003; Conway, 2005). In line with previous studies, our results indicate that the retrieval of immersive 3D-360° experiences differs from the retrieval of conventional 2D laboratory events (Schöne et al. 2016; Schöne et al. 2019; Kisker et al., 2019b). Hence, the well-established theta old/new effect does not seem to be unrestrictedly applicable to VR experiences. It might rather serve as an index for cue-matching of previously exogenously processed pictorial stimuli: Experiences encoded in the laboratory are recalled and visually matched to the test stimuli, but are not inevitably associated with the vivid and multimodal character of autobiographical memories and thus, might not provide a holistic representation of real-life mnemonic processing.

The question remains, which processes change their mode of operation in response to the recall of VR experiences. The theta old/new effect is predominantly associated with retrieval success (e.g. Nyhus & Curran, 2010). However, the VR and the PC group were likewise successful in the recognition task. As above mentioned, frontal-midline theta is associated with autobiographical mnemonic processing, but also regarded as an index for top-down control of memory retrieval (Klimesch et al., 1997b; Nyhus & Curran, 2010). Specifically, early theta-band increases indicate an attempt or the effort demands to retrieve engrams rather than successful retrieval per se (Klimesch et al., 2001a; Nyhus & Curran, 2010). Several studies investigating memory retrieval in general as well as the classical old/new effect in particular, explicitly differentiate retrieval effort and retrieval success (Klimesch et al., 2001a; Nyhus & Curran, 2010; Rugg et al., 1998; Konishi, Wheeler, Donaldson & Buckner, 2000). In particular, processes exclusively associated with retrieval success are engaged only if an attempted retrieval is successful. In contrast, retrieval effort refers to those processes engaged during a retrieval attempt per se, for example in recognition tasks, regardless of whether this attempt is successful or not (Rugg, Fletcher, Frith, Frackowiak & Dolan, 1996). Accordingly, the absence of a difference in memory success does not rule out that the effort required to achieve the very same retrieval outcome may vary.

Hence, the difference in the theta-band response to old pictures between the VR condition and the PC condition could reflect the two types of retrieval differing with respect to their effort demands (Conway, 1996; Haque & Conway, 2001; Conway & Pleydell-Pearce, 2000). Immersive VR experiences as part of an extensive autobiographical associative network (PBM, Schöne et al., 2019) can be effortless and, most of all, directly retrieved. In contrast, the retrieval of conventional stimuli triggers the iterative verification process and the suppression of irrelevant information, thus coming in with higher effort to recall memories. Direct retrieval of autobiographical memory is based upon a pronounced and stable memory pattern (Conway & Pleydell-Pearce, 2000) and enables spontaneous recall, which is rather automatic and effortless (Conway & Pleydell-Pearce, 2000 as cited in Willander & Larsson, 2007). It thus allows immediate recall of a cued memory. Generative or strategic retrieval of conventional stimuli, as observed in the PC condition, relies on central control of memory recall (Willander & Larsson, 2007). To verify the cued memory, irrelevant information has to be suppressed, while mental representation and cue are matched (Norman & Bobrow, 1979; Conway, 1996; Burgess & Shallice, 1996).

This interpretation of a visually guided matching process gains further support from the difference in posterior alpha oscillations, associated with visual processing (e.g. Clayton et al., 2018). Matching mental representation and cue is reflected by a generally reduced posterior alpha amplitude in the PC condition compared to the VR condition. This reduced alpha amplitude, commonly regarded as cortical activity (e.g. Berger, 1929 as cited in Klimesch et al., 1997b), on the one hand reflects elevated attention (e.g. Klimesch, et al. 1997a; Fries, Womelsdorf, Oostenveld & Desimone, 2008) and, on the other hand, successful suppression of irrelevant information (Sauseng et al., 2009; Jensen & Mazaheri, 2010). Especially, the co-occurrence of higher frontal theta responses and posterior alpha activity has been interpreted as a response to higher cognitive load, with 2D environments exhibiting higher cognitive load as compared to 3D environments (Dan & Reiner, 2017). Theta and alpha oscillations thus provide evidence for effortless and direct retrieval of immersive VR experience and a, in comparison, effortful and strategic retrieval of conventionally presented stimuli.

Nevertheless, the finding that the retrieval mechanisms underlying VR experiences and conventional laboratory experiences differ, does not invalidate previous well-established knowledge gained from conventional setups. Rather, it complements the immense insights from previous studies and demonstrates the delicate balance between high experimental control and ecological validity. Thus, controlled laboratory studies provide the foundations for understanding the complex mechanisms of human memory and are substantial for developing models. As a further refinement of these foundations, VR settings facilitate the transfer of experimental findings to everyday life and thus improve their generalizability and practicability.

## Conclusions

As a conclusion, we replicated the well-established theta old/new effect in a conventional laboratory setting, manifested in relative theta-band synchronization for old, and relative desynchronization for new stimuli. However, this effect could not be replicated for the immersive VR condition: Theta-band responses were equal for old and new stimuli. Hence, the canonical theta old/new effect might not be unrestrictedly applicable to VR experiences and thus, might not provide a holistic representation of real-life processes. Accompanied by higher alpha activity as compared to the VR condition, the theta-band synchronization in the PC condition might rather reflect higher retrieval effort than retrieval success per se. In contrast to laboratory events, memories obtained from VR experiences are spontaneous and effortless retrieved. Additionally, participants of the VR condition reported a higher sense of presence, which might enhance the self-relevance of the VR experiences. Crucially, self-referential processing and a facile, effortless recall are characteristic of autobiographical memory. Therefore, the effortless recall of VR experiences might approximate real-life memory more closely as compared to memories obtained from the laboratory. However, the VR group did not perform better in the memory test, as former research suggested. Hence, our results suggest that the memory processes underlying VR experiences are qualitatively different from conventional laboratory experiences, but under which conditions VR leads not only to altered mechanisms but also to a better memory performance compared to conventional settings should be the subject of further research.

## Electronic supplementary material

Below is the link to the electronic supplementary material.

Supplementary material 1 (PDF 30 kb)

## Data Availability

The datasets generated and analyzed during the current study are available in the Open Science Framework (OSF) repository, https://osf.io/q924w/?view_only=cc107f7a927f472e8e68e85aaa059e97. Stimulus material was obtained from the Library for Universal Virtual Reality Experiments (luVRe, https://www.psycho.uni-osnabrueck.de/fachgebiete/allgemeine_psychologie_i/luvre.html) and can be accessed upon request.
