# Supplementary material for: Virtual reality experiences promote autobiographical retrieval mechanisms: Electrophysiological correlates of laboratory and virtual experiences
Source: Psychol Res. 2020 Sep 15;85(7):2485–501. doi: 10.1007/s00426-020-01417-x (PMC8440245; doi:10.1007/s00426-020-01417-x)
Supplement: Supplementary file 1 — Supplementary material 1 (PDF 30 kb) [file 426_2020_1417_MOESM1_ESM.pdf]

Description of stimulus material (from Library of universal virtual reality experiments (luVRe))

accessed via https://www.psycho.uni-osnabrueck.de/fachgebiete/allgemeine_psychologie_i/luvre.html

luvre code	class	content
x_0101.mp4	target	View on canal in Amsterdam (tourist attraction)
x_0106.mp4	target	a public toilette
x_0202.mp4	target	Reticulated python in an aquarium
x_0301.mp4	target	Gym of a hotel (without people)
x_0311.mp4	target	Hotel room with guests sitting on a couch
x_0504.mp4	target	Prison cell in a museum
x_0702.mp4	target	Cows in a barn
x_0803.mp4	target	Technical instruments in an atomic shelter
x_1005.mp4	target	View on a lake in autumn
x_1202.mp4	target	Soccer training
x_1603.mp4	target	Boars in enclosure
x_6501.mp4	target	Choir sings in a cathedral in presence of an audience
x_1607.mp4	target	Elbe Philharmonic Hall (outerior, Hamburg)
x_1705.mp4	target	Showact: Groups of boys dancing breakdance (1)
x_2203.mp4	target	Shop for motorcycles and equipment
x_2312.mp4	target	A man is treated by an oculist
x_2314.mp4	target	Paramedics carry a woman on a gurney
x_2602.mp4	target	Pole dancing woman
x_2701.mp4	target	Original prision cells (without prisoners)
x_2705.mp4	target	Staircase with suicide prevention in a prision
x_2801.mp4	target	Horse riding
x_2901.mp4	target	Slide in a swimming centre
x_3001.mp4	target	A group of men stripping (show with large audience)
x_3104.mp4	target	Inside Carmen de los Mártires (Granada, Spain)
x_3201.mp4	target	Hotel bar with guests
x_3307.mp4	target	Hotel corridor without guests
x_3313.mp4	target	Kitchen in a hotel suite
x_3503.mp4	target	Dental treatment room
x_3603.mp4	target	Tuco-tuco (rat species: Ctenomys, Ctenomyidae) in an enclosure
x_3609.mp4	target	Arctic foxes looking for food in enclosure

x_4223.mp4	target	Red carpet of a prom night
x_4303.mp4	target	Man in a cave
x_4405.mp4	target	Tattooist tattoos a young woman at her ankle
x_5503.mp4	target	A group of young people searches and examines a room
X_4602.mp4	target	Two women ordering doner kebap in a fast food restaurant
x_4702.mp4	target	People entering and leaving a cinemas' salesroom
x_4801.mp4	target	Christmas party of a university department (Osnabrück University, Department of Psychology)
x_4903.mp4	target	Puppies playing
x_5001.mp4	target	Sparsely lit mine tunnel
x_5102.mp4	target	Funeral room with a laid coffin (empty)
x_5202.mp4	target	Minigolf in a black light course
x_5303.mp4	target	View on shelves in a supermarket without people)
x_5402.mp4	target	Interior view of a church steeple
x_5602.mp4	target	Young pianist playing
x_5702.mp4	target	view of an approaching helicopter
x_5802.mp4	target	A group of young women doing squats and lunges
x_5903.mp4	target	Two persons shooting at targets in a training facility
x_6001.mp4	target	Traffic at the train station
x_6303.mp4	target	The karate fighters surround the camera during training and shout fiercely
no code assigned	target	A screaming man runs into the room, pointing a gun at the camera
x_0105.mp4	distractor	dark interior of a building in Amsterdam
x_0306.mp4	distractor	Restaurant of a hotel (without people)
x_0405.mp4	distractor	Car in a dark car park
x_0701.mp4	distractor	Aviary with doves
x_0706.mp4	distractor	Horse in an indoor stable
x_0802.mp4	distractor	Switchboard in an atomic shelter
x_0901.mp4	distractor	People partying in a disco
x_1007.mp4	distractor	Field path
x_1102.mp4	distractor	Fire engines driving out of the garage
x_1303.mp4	distractor	Museum: Miniature Wonderland
x_1501.mp4	distractor	Woman spraying graffiti on the wall of an abandoned building
x_1605.mp4	distractor	Deers in an enclosure
x_1609.mp4	distractor	Hamburg's "Landungsbrücken" (bridge)

x_1613.mp4	distractor	Willkomm-Höft (Ship Greeting Station Welcome Point in Hamburg, Germany)
x_1615.mp4	distractor	Woman singing and playing the guitar
x_1910.mp4	distractor	Kart race with view from the grandstand
x_2001.mp4	distractor	Artist working on his artwork (constructed from yarns)
x_2003.mp4	distractor	Art installation in an art galery
x_2109.mp4	distractor	Group of MMA fighters doing a battle shout
x_2201.mp4	distractor	Parking lot for motorbikes
x_2301.mp4	distractor	Trauma room in an emergency department (without people, no blood or injuries visible)
x_2304.mp4	distractor	Ward room in an emergency department (without people, no blood or injuries visible)
x_2317.mp4	distractor	Ambulance arrives at the hospital
x_2703.mp4	distractor	Exterior view on prison cells
x_2704.mp4	distractor	Shower room
x_2903.mp4	distractor	Free fall slide in a swimming centre, people are riding it
x_3103.mp4	distractor	Courtyard of Carmen de los Mártires (Granada, Spain)
x_3105.mp4	distractor	Inner yard of Carmen de los Mártires
x_3107.mp4	distractor	Market place at night
x_3304.mp4	distractor	Winery without guests
x_3310.mp4	distractor	Empty room of a hotel
x_3401.mp4	distractor	Wien City Prater
x_3606.mp4	distractor	Penguins in enclosure
x_3701.mp4	distractor	Motorway bridge
x_3802.mp4	distractor	People bouldering
x_3903.mp4	distractor	Interior of a luxurious campervan
x_4001.mp4	distractor	Witness stand of a courtroom
x_4101.mp4	distractor	Wodden attic of an ancient mill
x_4220.mp4	distractor	Opening dance during a prom night
x_4221.mp4	distractor	Outerior of Osnabrück Halle (building in Osnabrück, Germany)
X_4502.mp4	distractor	Ferris wheel going upwards, one person in the cabine (Osnabrück, Germany)
X_4704.mp4	distractor	Interior of a dimly lit bar with flickering lights
x_5401.mp4	distractor	View over the city of Osnabrück from the church steeple
x_5904.mp4	distractor	An elderly man shooting with a rifle at targets
x_5906.mp4	distractor	People sitting in a club bar
x_6003.mp4	distractor	A train leaving the station
x_6101.mp4	distractor	A young man's hair is cut.

x_6201.mp4	distractor	A mixed group trains karate
x_6402.mp4	distractor	Interior view of a laser tag arena during the game
no code assigned	distractor	A couple ist discussing fiercely
